# Early MRI predictors of prognosis in multiple sclerosis

**DOI:** 10.1007/s00415-019-09589-2

**Published:** 2019-11-08

**Authors:** Nikunj Davda, Emma Tallantyre, Neil P. Robertson

**Affiliations:** 1 Department of Neurology, University Hospital of Wales, Heath Park, Cardiff, C14 4XN UK; 2 Department of Neurology, Institute of Psychological Medicine and Clinical Neurosciences, Cardiff University, Cardiff, C14 4XW UK

## Introduction

Although some simple informative early clinical markers of disease course are available, individual prognosis in multiple sclerosis (MS) remains unreliable and leads to difficulties both in counseling and treatment of newly diagnosed patients. Decisions to initiate immunomodulatory treatment often depend on recent inflammatory disease activity, whereas accurate personalised long-term prognosis, which might inform both the timing and type of treatment, remains elusive. This month’s journal club reviews three studies that examine the predictive value of early MRI features in determining selective long-term outcomes such as future physical and cognitive disability and transition to secondary progressive disease phase. The use of brain imaging to visualise both cortical and chronically active ‘smouldering’ white matter lesions, may reduce the clinical-radiological paradox that has previously been described in MS. The studies outlined below explore some conventional and more novel MRI markers at baseline for their ability to predict subsequent disease progression and prognosis.

## Early imaging predictors of long term outcomes in relapse-onset multiple sclerosis

This study aimed to determine which early MRI features best predict future physical and cognitive disability, including conversion to secondary progressive MS (SPMS) at 15 years, in a cohort of relapse onset MS patients. Patients between the ages of 16 and 50 presenting to two specialist London hospitals with a clinically isolated syndrome (CIS) and no relevant previous neurological history were recruited (*n* = 178). Patients underwent MRI scans at baseline, 1 year and 3 years to document intracerebral, spinal cord T2 lesions, gadolinium-enhancing lesions and total brain volume. They were then followed up at 15 years to assess disease course (either relapsing or SPMS), physical disability (expanded disability status scale (EDSS), timed 25 foot walk and 9 hole peg test) and cognitive disability (paced auditory serial addition test and single digit modalities test).

In multivariate models, SPMS at 15 years was best predicted by a baseline scan demonstrating ≥ 2 gadolinium-enhancing lesions or ≥ 1 spinal cord lesion (odds ratio 3.16 and 4.71), or a 1-year scan demonstrating ≥ 1 new lesion (compared with baseline) in either the spinal cord or infratentorial compartment (odds ratio 5.72 and 7.02). The use of 3-year data added further weight to the predictions: in people with no new spinal or infratentorial lesions over the first 3 years after CIS the estimated risk of secondary progressive multiple sclerosis after 15 years was 0.9% (95% CI 0, 2.7%), compared with 53.1% (31.7, 74.6%) in patients with ≥ 1 new spinal and ≥ 1 new infratentorial lesions at 3 years.

Early MRI lesions were also positively correlated with EDSS at 15 years, particularly gadolinium-enhancing and spinal cord lesions at baseline, and enhancing lesions at 1 and 3 years. The authors conclude that early focal inflammatory activity and particularly spinal cord lesions predict long term outcomes in MS, including the transition to SPMS.

*Comment* This study measured baseline and early interval change in brain and spinal cord MRI appearance in a large CIS cohort, and modeled the predictive value of lesion appearances and location for long-term physical and cognitive outcomes. While many investigators are seeking novel biomarkers to use in MS, we ought not to forget that we do have some valuable tools already available to us in the clinic for predicting outcome. While many centres are trying to limit the use of gadolinium for follow-up scans, here we see another reason why baseline scanning with contrast is valuable in MS. One of the uncertainties of this study is the potential confounding effect disease modifying therapies (DMTs) may have had on outcomes. Since the cohort was recruited between 1994 and 2004, we might assume that the early imaging findings may have predated the widespread use of DMTs; later use of DMT may have influenced outcomes. These data will add value by increasing the information available to patients and clinicians at disease presentation, and should probably be factored into analyses of more novel prognostic biomarkers.

*Brownlee WJ et al. (2019) Brain 142:2276–87*

## Longitudinal characterisation of cortical lesion development and evolution in multiple sclerosis

Post mortem histological examination of the brain demonstrates that cortical demyelination is often more extensive than white matter demyelination in MS. However, cortical demyelination is not well visualized using conventional MRI techniques. This study used ultra high field (7T) MRI to explore the relationship between cortical lesion and white matter lesion accrual and to determine contribution of cortical and white matter lesion load to cortical thickness and disability. Adults between the ages of 18 and 65 who had a diagnosis of clinically defined multiple sclerosis and were stable either on or off DMTs were recruited to between 2010 and 2016. Patients underwent two interval 7T MRI scans to assess burden and distribution of cortical lesions and also 3T MRI to estimate cortical thickness. Disability progression was defined by a sustained increase in EDSS.

People with SPMS demonstrated higher cortical and white matter lesion counts and volumes compared to those with RRMS. Moreover, the rate of cortical lesion accumulation was higher in people with RRMS who developed SPMS than in those who remained in RRMS (3.6 lesions/year vs. 1.1 lesions/year respectively). Interestingly, the overall rate of accumulation of cortical lesions appeared both higher than, and independent of the accumulation of white matter lesions. Cortical lesion burden at baseline (but not white matter lesion burden) was found to correlate with baseline EDSS and to predict subsequent EDSS change. Cortical lesions were widely distributed across lobes but displayed a slight predominance for the sulci in RRMS.

*Comment* This longitudinal study used an ultra high-field MRI marker to predict subsequent disability outcomes in MS. Since 7T MRI is not available to most clinicians, this has limited immediate clinical utility, but serves as an important reminder that conventional MRI is insensitive to some clinically relevant pathology in MS. Given the prevalence of grey matter demyelination in MS histopathology, improved MRI visualization, facilitating increased understanding of its clinical relevance, may prove to be important areas of future research.

*Treaba CA et al. (2019) Radiology 291:740–9*

## Chronic white matter lesion activity predicts clinical progression in primary progressive multiple sclerosis

Histological examination of MS lesions reveals an important subgroup of lesions with ‘smouldering’ inflammatory activity, which is more notable in people with progressive MS, and may account for some of the gradual disability experienced by this group. Some T2 hyperintense lesions are observed to slowly expand over time, and some demonstrate greater T1 hypointensity, an imaging feature known to correlate with greater axonal damage. This study used imaging data acquired during the ORATARIO trial; the phase 3 study of ocrelizumab versus placebo in people with primary progressive multiple sclerosis (PPMS), to explore the contribution of MS lesion characteristics to clinical outcomes.

Key eligibility criteria for the ORATARIO trial were: age 18–55, diagnosis of PPMS, and EDSS 3–6.5. The placebo group demonstrated an increase in T2 lesion volume from baseline over 120 weeks, but the overall increase in T1 hypointense lesion volume was more than twice that of increase in T2 lesion volume. Slowly expanding lesions accounted for a small proportion of lesions overall, but a high amount of T1 lesion volume accumulation. This suggests that smouldering activity within chronic lesions, rather than the appearance of new lesions, accounts for the majority of the increase in T1 hypointense lesion volume.

Chronic lesion activity (the accumulation of T1 hypointensity within existing lesions) was the strongest predictor of disability outcomes, rather than brain volume loss or the rate of accumulation of new T1 or T2 lesions. Participants in the ocrelizumab arm had a lower rate of T1 and T2 lesion accumulation than those receiving placebo. Encouragingly, an ocrelizumab treatment effect was seen with regards to reduced accumulation of T1 hypointensity, both in new and existing lesions. The study authors concluded that increase in T1 lesion burden was largely due to smouldering disease activity within chronic lesions, and that this imaging finding was the best predictor of disease progression.

*Comment* This is a large, prospective imaging dataset from over 500 people with PPMS, where it was possible to characterise MS lesions according to T1 and T2 tissue contrast as well as whether ot not lesions were slowly expanding. This revealed novel insights into the likely mechanism of accumulation of neuroaxonal pathology in progressive MS, but also brought encouragement that contemporary therapies may slow the accumulation of axonal pathology. The study did not incorporate data on the anatomical location of lesions, which may have additional value in predicting outcome. For example, no spinal MRI data were collected, which may be highly relevant in this patient cohort. The confirmation that smouldering lesions, which may have a different and unique pathology to acute lesions possibly with persistent inflammatory activation, may contribute significantly to disease burden in this patient group is of considerable interest and may prompt a future change in research and therapeutic direction.

*Elliot C et al. (2019) Brain 142:2787–99*

*Summary* All these studies provide valuable observations on underlying pathology, often not apparent in conventional imaging techniques and below the surface of what we might consider a ‘standard assessment’. In addition, together with clinical features, they increasingly raise the prospect of a more individualised approach to patient management and counselling and start to explain the reasons for the well-described clinical-radiological paradox as well as the importance of grey matter pathology.

